# Hepcidin is upregulated and is a potential therapeutic target associated with immunity in glioma

**DOI:** 10.3389/fonc.2022.963096

**Published:** 2022-09-27

**Authors:** Tianyu Dong, Bo Zhang, Runjiao Zhang, Chang Wang, Xiaopeng Liu, Fei Wang, Nana Hao, Ke Tan, Yan-Zhong Chang

**Affiliations:** ^1^Key Laboratory of Molecular and Cellular Biology of Ministry of Education, Key Laboratory of Animal Physiology, Biochemistry and Molecular Biology of Hebei Province, College of Life Sciences, Hebei Normal University, Shijiazhuang, China; ^2^Department of Anatomy, Hebei Medical University, Shijiazhuang, China; ^3^Department of Neurosurgery, The Second Hospital of Hebei Medical University, Shijiazhuang, China; ^4^Department of Neurology, Handan Central Hospital, Handan, China

**Keywords:** hepcidin, glioma, prognostic biomarker, immune cell infiltration, iron

## Abstract

**Background:**

Glioma is the most common primary malignant brain tumor with high mortality and poor prognosis. Hepcidin is a fascinating iron metabolism regulator. However, the prognostic value of hepcidin HAMP in gliomas and its correlation with immune cell infiltration remain unclear. Here, we comprehensively elucidate the prognostic value and potential role of hepcidin in gliomas.

**Methods:**

Hepcidin gene expression and clinical characteristics in glioma were analyzed using the CGGA, TCGA, Rembrandt and Gravendeel glioma databases. A survival analysis was conducted using Kaplan–Meier and Cox regression analyses. A gene set enrichment analysis (GSEA) was conducted to select the pathways significantly enriched for hepcidin associations. The correlations between hepcidin and immune cell infiltration and immunotherapy were analyzed using network platforms such as CIBERSORT and TIMER.

**Results:**

In glioma tissues, the expression of hepcidin was significantly increased. High hepcidin expression is related to grade, age, PRS type, IDH mutation, chemotherapy status and 1p19q codeletion status, which significantly indicates the poor prognosis of glioma patients. Hepcidin can be used as an independent prognostic factor for glioma through the multivariate COX regression analysis. The results of Gene Ontology (GO), Kyoto Encyclopedia of Gene and Genome (KEGG) and gene set enrichment analysis (GSEA) indicated that hepcidin was involved in the immune response. In addition, hepcidin expression was positively correlated with the degree of immune cell infiltration, the expression of various immune cell markers and the efficacy of immunotherapy.

**Conclusion:**

Our results indicate that hepcidin can be used as a candidate biomarker to judge the prognosis and immune cell invasion of gliomas.

## Introduction

Glioma is a common primary malignant brain tumor and arises throughout the central nervous system. According to the World Health Organization (WHO) classification of central nervous system tumors in 2021, gliomas of grades I and II are classified as low-grade gliomas (LGGs) (1). LGGs have a tendency to relapse and transform into high-grade gliomas (WHO grades III and IV). The annual incidence of high-grade gliomas (HGGs) has recently increased, and these gliomas have high disability and mortality rates (2, 3). At present, many studies have shown that oxidative stress can drive mutations and accelerate the progression of gliomas when physiological iron metabolism is disturbed (4). Therefore, clarifying the relationship between iron-related genes and gliomas and identifying effective biomarkers and new treatment targets are helpful for individualized treatment and prognosis prediction.

Iron is involved in cell proliferation and differentiation in eukaryotic metabolism (5). Defective iron homeostasis is significantly involved in the development of human cancers, and the expression of iron homeostasis-related genes is dysregulated in tumors (6). Serum ferritin levels are elevated in patients with many types of cancer. Ferritin is expressed at high levels in prostate cancer cells and regulates cell proliferation, migration and apoptosis by increasing the intracellular iron content (7). High plasma ferritin levels block the circulation of iron from intestinal epithelial cells and macrophages (which contribute to anemia in patients with cancer) and may lead to the accumulation of iron in tumor cells through the degradation of ferroportin (FPN1), resulting in the activation of signaling pathways, such as Wnt (8) and NF-κB (9), which contribute to tumor progression. In addition to the ferritin synthesized by the liver, cancer cells also synthesize ferritin. Upregulated expression of TFRC was markedly correlated with a poor prognosis for patients with breast cancer, indicating that TFRC may be an independent prognostic marker for breast cancer (10). Moreover, TFRC is involved in the immune response and immune cell infiltration (10). In contrast, ceruloplasmin (CP) expression is downregulated in breast cancer (11). Higher CP expression is related to shorter overall survival (OS) of patients with breast cancer (11). Additionally, the expression of the only known mammalian iron exporter, FPN1, is decreased in lung cancer and low FPN1 expression corresponds to a worse prognosis for patients with lung cancer (12). Interestingly, FPN1 was identified as a suppressor of ferroptosis and its expression is closely correlated with the prognosis of patients with glioblastoma (GBM) and is associated with immunosuppression (13).

In glioma, iron metabolism has recently become a therapeutic target and potential prognostic marker (14, 15). An increasing number of studies has revealed the close relationship between high-grade gliomas and iron metabolism. Iron accumulation promotes the proliferation of glioma cells. Patients with high-grade gliomas have higher levels of serum ferritin (16). Hypoxia-induced ferritin light chain expression is also involved in the epithelial–mesenchymal transition (EMT) and chemotherapy resistance in high-grade gliomas (17). Numerous studies have shown that hepcidin-FPN signaling plays a key role in regulating iron metastasis and tumor growth (18). During iron overload, the expression of hepcidin (HAMP) increases, which interacts with FPN1 to promote its internalization and degradation in lysosomes (19). Hepcidin, an antimicrobial peptide rich in cysteine, consists of 20, 22 and 25 amino acid peptides (20). Hepcidin expression is elevated in patients with lung cancer. Increased hepcidin expression is associated with various clinical parameters and predicts a worse prognosis for patients with lung cancer (21). Meanwhile, hepcidin expression is also upregulated in kidney, testicular and gastric cancers (22, 23). Increased expression of hepcidin also represents a risk factor for these tumors. However, hepcidin expression is significantly decreased in liver cancer (24, 25). Downregulated expression of hepcidin is closely correlated with liver cancer aggressiveness, immune cell infiltration and worse survival outcomes of patients with liver cancer (25). A recent study indicated that hepcidin was also produced in colon tumor epithelium and correlated with shorter patient survival (26). Loss of hepcidin in the colonic epithelium significantly reduces the tumor number and size compared with littermate controls (26). Together, hepcidin may play a dual role in different types of tumors. The disorder of iron metabolism caused by the abnormal expression of hepcidin may be an important factor contributing to tumor invasion and metastasis.

Despite the close relationship between iron homeostasis and tumorigenesis, there is no study on the role and clinical significance of hepcidin in the pathogenesis and prognosis of gliomas. Thus, the purpose of this study was to combine a variety of bioinformatics methods to investigate whether hepcidin is involved in glioma, tumor metastasis and immune invasion and to explore its molecular mechanism. We found that the expression of hepcidin in glioma tissues was significantly upregulated compared with that in nontumor tissues. In addition, the expression of hepcidin increased with tumor grade. High hepcidin expression was negatively correlated with the prognosis of patients with glioma. In addition, the expression of hepcidin in gliomas was closely related to the infiltration of B cells, CD4+ T cells, CD8+ T cells, macrophages, neutrophils and dendritic cells. Single-cell RNA sequencing analysis revealed that hepcidin was expressed at high levels in macrophages. Importantly, hepcidin expression was significantly correlated with the tumor mutation burden (TMB), tumor microenvironment (TME), immunosuppressant targets and tumor immune dysfunction and exclusion (TIDE) scores. These observations emphasize the important role of hepcidin in tumorigenesis and suggest that hepcidin may play an important role in regulating immune cell infiltration and the response to immunotherapy of gliomas.

## Materials and methods

### Raw data collection

We obtained the RNA sequencing data of diffuse glioma patients from two independent datasets in the Chinese Glioma Genome Atlas (CGGA) database (http://www.cgga.org.cn): the CGGA Dataset 1 (n=325) and the CGGA Dataset 2 (n=693) (27). A dataset containing the gene expression profiles and clinical information of glioma patients (n=698) was downloaded from the publicly available The Cancer Genome Atlas (TCGA) database (28). The hepcidin expression information (FPKM normalized) and clinical data were extracted. The collected clinicopathological data, including gender, age, grade, survival status and survival duration (in days), were integrated, and the default value samples were deleted according to the statistical needs for the follow-up research.

### Analysis of the relationship between hepcidin expression and glioma grade

Through the Hiplot open-source network platform UCSCXenaShiny module (https://hiplot.com.cn/advance/ucscxena), we analyzed the expression data of hepcidin in 33 cancer types from the TCGA and grouped them according to normal or tumor tissues. hepcidin gene expression and clinical characteristics of glioma samples from the CGGA, TCGA, Rembrandt and Gravendeel glioma databases were obtained through the GlioVis network platform (http://gliovis.Bioinfo.cnio.es/).

### Tissue samples

One case for each WHO grade of human astrocyte-derived tumors resected by the Neurosurgery Department at the Second Hospital of Hebei Medical University from January 2019 to January 2020 was collected. All specimens were collected immediately after surgical tissue resection, stored directly in liquid nitrogen and then confirmed by pathology. Patients who received preoperative radiotherapy or chemotherapy were excluded from this study. The Ethics Committee of the Second Hospital of Hebei Medical University approved this study (Approval #: 2017-P035). The requirement for written informed consent to participate was waived by the Ethics Committee.

### Analysis of the relationship between hepcidin and the survival of glioma patients

To study the effect of hepcidin on the survival of glioma patients, we divided the samples from various glioma databases into a high-expression group and a low-expression group according to the median hepcidin expression level. Kaplan–Meier survival curves were plotted, and log-rank and Wilcoxon tests were performed based on the survival data of glioma patients from the CGGA, TCGA, Rembrandt and Gravendeel databases. Events of the high- and low-expression groups in each database were summarized, and then the relative survival risk ratio of each group in each database was calculated by using the meta-analysis of the Binary Data tool on the Hiplot network platform, and forest maps of the survival risk factors for patients were drawn for each database.

### The relationship between the hepcidin expression level and clinical traits in glioma

First, we analyzed the relationships between hepcidin expression and age, sex, isocitrate dehydrogenase (IDH) mutation, glioma subtype, 1p19q codeletion status and other clinical features of CGGA and TCGA data and drew a heatmap using the R package “ComplexHeatmap”. Then, based on the CGGA dataset, we analyzed the differences in each clinical feature in the high and low hepcidin expression groups. Univariate and multivariate independent prognostic analyses of the CGGA and TCGA datasets using R were performed to further observe the relationship between hepcidin expression and the prognosis. Next, we constructed a nomogram model and calibrated it with R language. The nomogram model had a better performance in predicting prognosis than the traditional staging system. Furthermore, the 1-year, 3-year and 5-year ROC curves of CGGA and TCGA datasets were drawn using the R language to determine the accuracy of hepcidin expression in predicting patient survival.

### Analysis of hepcidin-interacting genes and proteins

The GeneMANIA database (http://www.genemania.org) was used to construct the hepcidin interaction network. The STRING online database (https://string-db.org/) was used to construct a protein–protein interaction (PPI) network of hepcidin. The correlations between hepcidin and iron metabolism-related genes were investigated based on the TCGA-LGG and TCGA-GBM databases.

### Functional analysis of differentially expressed genes between the high and low hepcidin expression groups in the CGGA and TCGA datasets

The genes coexpressed with hepcidin in the CGGA and TCGA databases were identified by performing a coexpression analysis. The R packages “corrplot” and “circlize” were used to draw circle graphs of representative genes that were significantly related to hepcidin. CGGA and TCGA samples were divided into two groups according to the expression of the hepcidin gene. The differentially expressed genes (DEGs) between the two groups were screened by the “limma” package of R language, and a heatmap was drawn by the “pheatmap” package. Furthermore, to clarify the biological functions and pathways hepcidin is involved in, R language was used for Gene Ontology (GO) and Kyoto Encyclopedia of Genes and Genomes (KEGG) analyses of the DEGs in the CGGA database. The c5.go.v7.4.symbols and c2.cp.kegg.v7.4.symbols datasets were used for gene set enrichment analysis (GSEA) of hepcidin. For each analysis, 1000 repetitions of gene set permutations were performed. The phenotype label was the expression level of hepcidin. Additionally, the nominal (NOM) P value and normalized enrichment score (NES) were calculated to sort the enriched pathways in each phenotype. Gene sets with NOM P value and false discovery rate (FDR) q value ≤ 0.05 were considered significantly enriched gene sets.

### Analysis of the relative abundance of tumor-infiltrating immune cells

CIBERSORT (http://cibersort.stanford.edu/), a deconvolution algorithm based on gene expression, has been widely used to analyze the correlation between gene expression in tumors and TIICs. According to the gene expression profile of complex tissues, this analysis can be used to characterize the heterogeneity of cells. CIBERSORT can recognize immune cell types sensitively and accurately, so they can be further analyzed. Based on the deconvolution algorithm, we downloaded the gene annotation matrix of 22 immune cell subtypes provided by the CIBERSORT network platform and calculated the P value for each sample in the CGGA and TCGA datasets. At the same time, the correlation between immune cells in each dataset was visually presented by R language. CIBERSORT was used to output the composition of infiltrating immune cells in each sample. Therefore, the relative proportions of various immune cells in each group were compared effectively and were visualized as a box diagram. According to the correlation between immune cells and hepcidin, we drew a lollipop map of immune cell correlations. The correlation of hepcidin and immune cell infiltration in LGG and GBM was analyzed in TIMER. The “Gene” module can investigate the relationship between hepcidin expression and immune cell infiltration levels (B cells, CD8+ T cells, CD4+ T cells, neutrophils, macrophages, and dendritic cells) using the TCGA database. TIMER was also applied to investigate the relationship between hepcidin expression and different gene marker sets of immune cells by using the “Correlation” module. The correlations of hepcidin expression with immune infiltration were evaluated by purity-correlated partial Spearman’s correlation and statistical significance.

### Analysis of the correlation between hepcidin expression and immunity

According to the median expression level of hepcidin, the samples were divided into high- and low-expression groups. Using the R language tool, we analyzed the expression of hepcidin in relation to the TME and TMB of the two groups. The TME scores were compared between the two groups in the CGGA database and visualized by a violin map. TMB refers to the number of genes with mutations per 1 million bases. We downloaded and sorted the mutation data of glioma through the TCGA, calculated and compared the difference in TMB between the two groups in the TCGA glioma dataset by R language, and analyzed the correlation between hepcidin expression and TMB. TIMER (https://cistrome.shinyapps.io/timer/), an interactive web portal, can perform a comprehensive analysis of the infiltration levels of different immune cells. In the present study, hepcidin expression in multiple types of cancer was evaluated through the “Diff Exp” module. Drugs targeting the immune checkpoint inhibitors of PD-1, PD-L1 and CTLA-4 are increasingly being shown to benefit cancer patients. Additionally, the relationships between hepcidin and PD-1, PD-L1 and CTLA-4 levels were determined by calculating Spearman’s correlation coefficients using the “correlation analysis” module of the GEPIA web portal (http://gepia.cancer-pku.cn/index.html).

### Single-cell RNA sequencing analysis

Single-cell RNA sequencing analyses were conducted using the Tumor Immune Single-cell Hub (TISCH) and scTIME Portal databases. TISCH (http://tisch.comp-genomics.org/home/) is a comprehensive web resource which provides interactive gene expression visualization and characterization of the TME at the single-cell level. GSE84465, GSE89567, GSE131928, GSE148842 and GSE138794 datasets were analyzed using the TISCH database. The scTIME Portal (http://sctime.sklehabc.com/unicellular/home) is an online single-cell RNA sequencing analysis database for single-cell transcriptomes of the tumor immune microenvironment. GSE138794 and GSE131928 datasets were analyzed using the scTIME Portal database.

### Immunohistochemical staining

The paraffin-embedded tissue was heated for 90 min in an oven at 60°C. The slides were then hydrated in ethanol and xylene at different concentrations (100%, 95% and 75%). Then, 3% H2O2 was added and incubated at room temperature for 10 min after washing with PBS for three times. Then, the slides were completely immersed in 95°C antigen repair solution for 10 min and cooled naturally. After washing with Triton-PBS (100x), the cells were sealed with 1% bovine serum albumin for 30 min. Then, the primary antibody was added and incubated overnight at 4°C. On the second day, the slides were washed with PBS (3×10 min/wash) and then incubated with a secondary antibody for 1 hour. DAB was added to the slides, and the color reaction was stopped with tap water. Hematoxylin was repeatedly stained for 1 min and separated with 1% hydrochloric acid ethanol solution. Finally, the slides were covered with a neutral essence, and images were taken with an Olympus microscope (Tokyo, Japan).

### Statistical analysis

The data are expressed as the mean ± standard deviation (SD). Significant differences between the means ± the standard deviations of two different groups were examined using Student’s t test; one-way ANOVA was used for more than two groups. Spearman correlation analysis was used for correlation analysis. The patients were divided into high- and low-expression groups according to the 50% cutoff point of gene expression. Differences in survival between groups were evaluated *via* Kaplan–Meier survival analysis with a log-rank significance test. Cox regression models were applied to identify prognostic factors. GraphPad Prism 8.0 software (GraphPad Inc., San Diego, CA, USA) was used to generate the graphs. A *p* value less than 0.05 was considered statistically significant.

## Results

### The expression of hepcidin increased with increasing tumor grade

**Figure 1:**
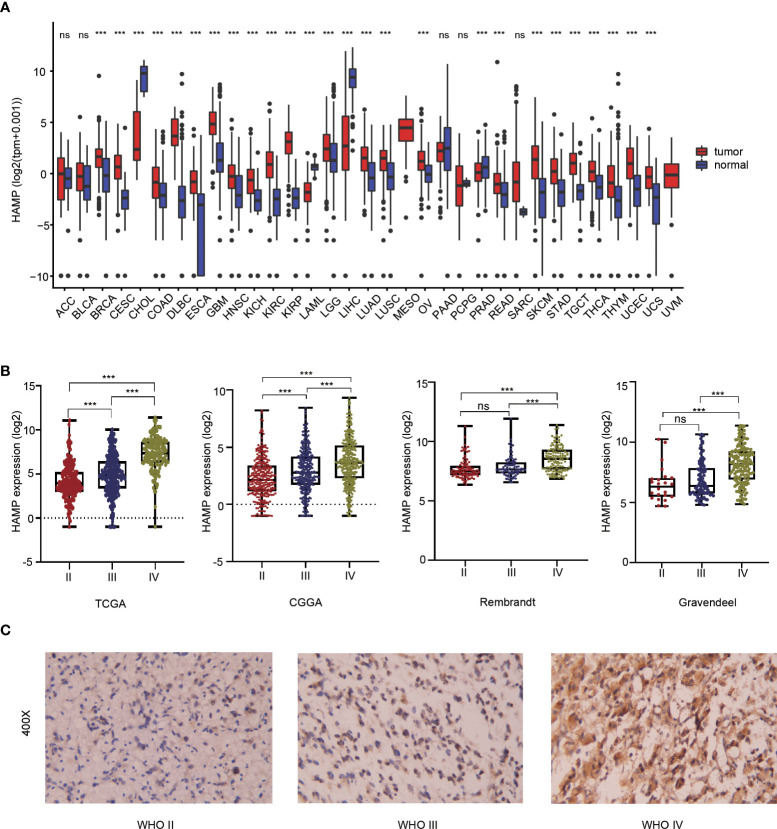
Analysis of the relationship between hepcidin expression and glioma grade. **(A)** Pan-cancer analysis of hepcidin expression in the TCGA database. **(B)** Relationship between the expression level of hepcidin and grade of glioma. **(C)** IHC staining of tissues with different glioma grades. ***p < 0.001, ns, not significant..

Pan-cancer analysis showed that there were significant differences in hepcidin expression between multiple tumor tissues and adjacent tissues according to the TCGA and GTEx databases. The expression of hepcidin in GBM, LGG, lung adenocarcinoma (LUAD), breast invasive carcinoma (BRCA) and many other tumor tissues was higher than that in normal tissues **(** **Figure 1A****)**. To explore the expression level of hepcidin in different grades of glioma, we analyzed the relationship between the expression level of hepcidin and glioma WHO grade in the CGGA, TCGA, Rembrandt and Gravendeel glioma databases. These results showed that the expression of hepcidin was significantly upregulated with increasing glioma grade **(** **Figure 1B****)**. The histochemical staining images of glioma showed that the protein expression of hepcidin in high-grade gliomas was higher than that in LGGs **(** **Figure 1C****)**.

### Analysis of the relationship between hepcidin and the survival of glioma patients

**Figure 2:**
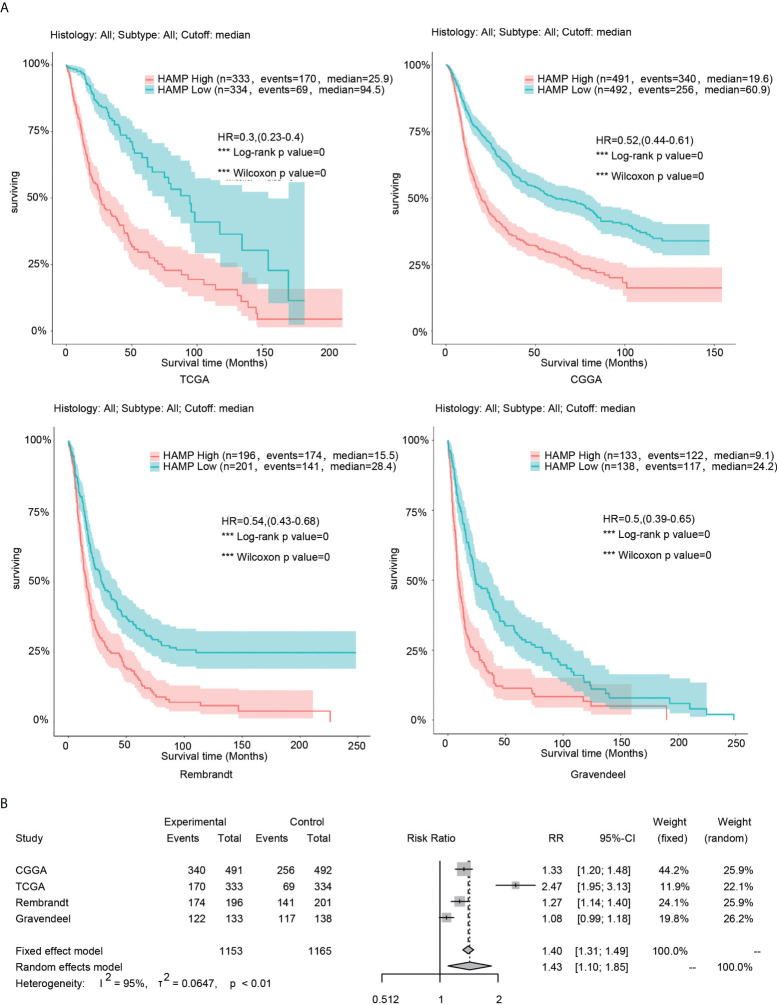
Analysis of the relationship between hepcidin and survival in glioma. **(A)** Kaplan–Meier plots of hepcidin in a variety of glioma datasets. The 95% confidence interval (CI) is shown. The patients were divided into high- and low-expression groups by the median expression level. **(B)** Forest plot of the risk ratio for patients with high hepcidin expression levels compared with patients with low hepcidin expression levels. ***p < 0.001.

In the four glioma databases (CGGA, TCGA, Rembrandt and Gravendeel), the samples were divided into low- and high-expression groups according to the median hepcidin expression. Kaplan–Meier curve analyses and statistical tests showed that in the four glioma datasets, the overall survival (OS) rate of patients with high hepcidin expression was significantly lower than that of patients with low hepcidin expression in glioma (*p* < 0.001) **(** **Figure 2B****)**. In view of the large sample size gap among the four cohorts, to improve the reliability of the results, the fixed effect model was used to estimate the risk ratio (RR) of the four glioma datasets for meta-analysis. The results showed that the OS time of patients with low hepcidin expression was longer than that of patients with high hepcidin expression (fixed effect model: RR: 1.40, 95% CI 1.31/1.49) **(** **Figure 2B****)**.

### Analysis of the relationship between hepcidin and clinical features in glioma

**Figure 3:**
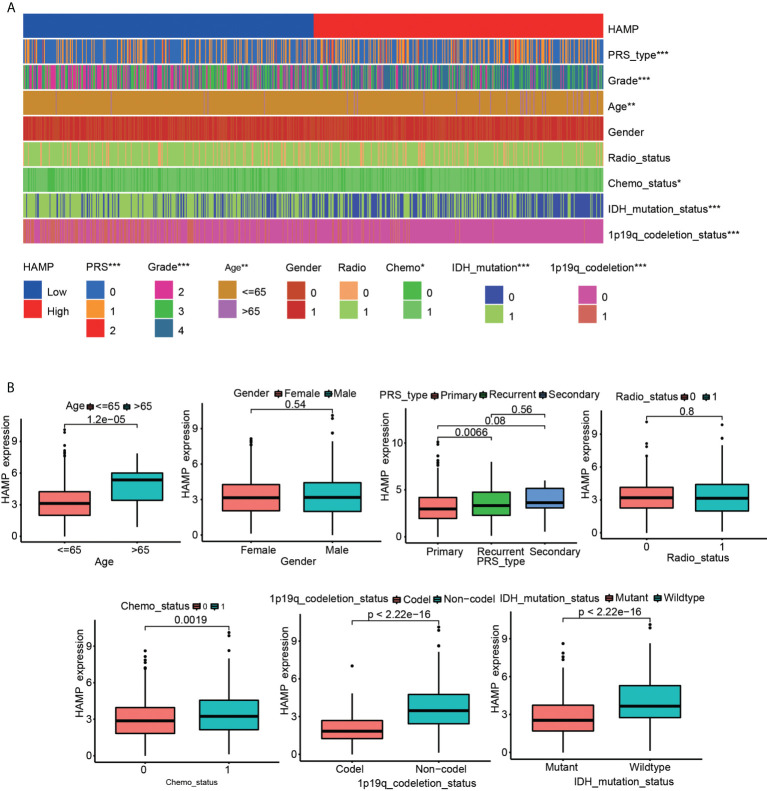
Analysis of the relationship between hepcidin expression and the clinical features of patients included in the CGGA glioma dataset. **(A)** The relationship between hepcidin expression and clinical features of patients in the CGGA dataset. **(B)** Each clinical feature was analyzed for differences of hepcidin expression in different groups. *p < 0.05, **p < 0.01, and ***p < 0.001.

**Figure 4:**
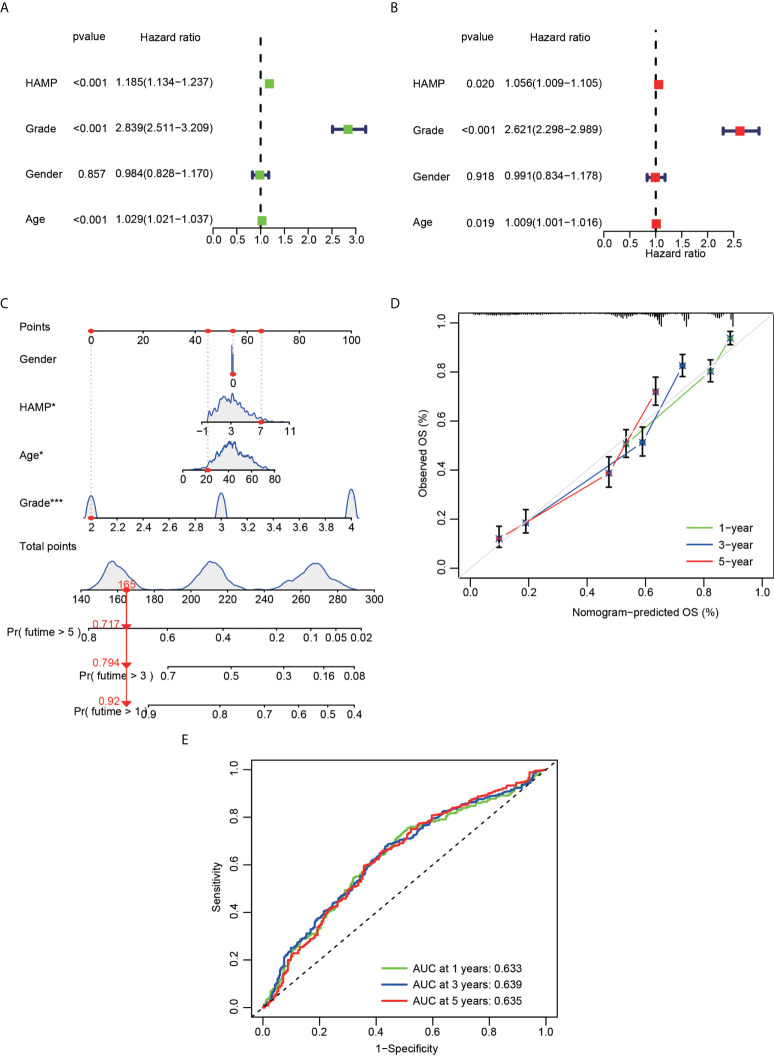
Cox regression analysis and establishment of a prognostic model using the CGGA dataset. **(A)** Univariate analysis of hepcidin expression in the CGGA dataset. **(B)** Multivariate analysis of hepcidin expression in the CGGA dataset. **(C)** The nomogram was constructed based on four factors for predicting 1-year, 3-year or 5-year survival of patients with glioma in the CGGA database. **(D)** The calibration plots of internal validation in CGGA dataset showed good consistency in predicting 1-year, 3-year or 5-year survival. **(E)** The 1-year, 3-year and 5-year ROC curves for the CGGA dataset. *p < 0.05 and ***p < 0.001.

Significant correlations were observed between hepcidin expression and the glioma grade, age, PRS type, IDH mutation, chemotherapy status, and 1p19q codeletion status in the CGGA dataset (*p* < 0.001) **(** **Figures 3A, B****)**. Significant correlations were observed between hepcidin expression and glioma grades and age in TCGA dataset (*p* < 0.001) **(** **Supplementary Figures S1A, B****)**. Univariate and multivariate Cox analyses showed that hepcidin, an individual variable, may be an independent prognostic factor in CGGA **(** **Figures 4A, B****)** and TCGA datasets **(** **Supplementary Figures S2A, B****)**. The nomogram model constructed based on hepcidin expression had good performance in predicting the prognosis of patients included in CGGA **(** **Figures 4C, D****)** and TCGA datasets **(** **Supplementary Figures S2C, D****)**. The areas under the ROC curves (AUCs) at 1 year, 3 years and 5 years in CGGA **(** **Figure 4E****)** and TCGA **(** **Supplementary Figure S2E****)** glioma datasets were all greater than 0.6.

### Identification of hepcidin-interacting genes and proteins

**Figure 5:**
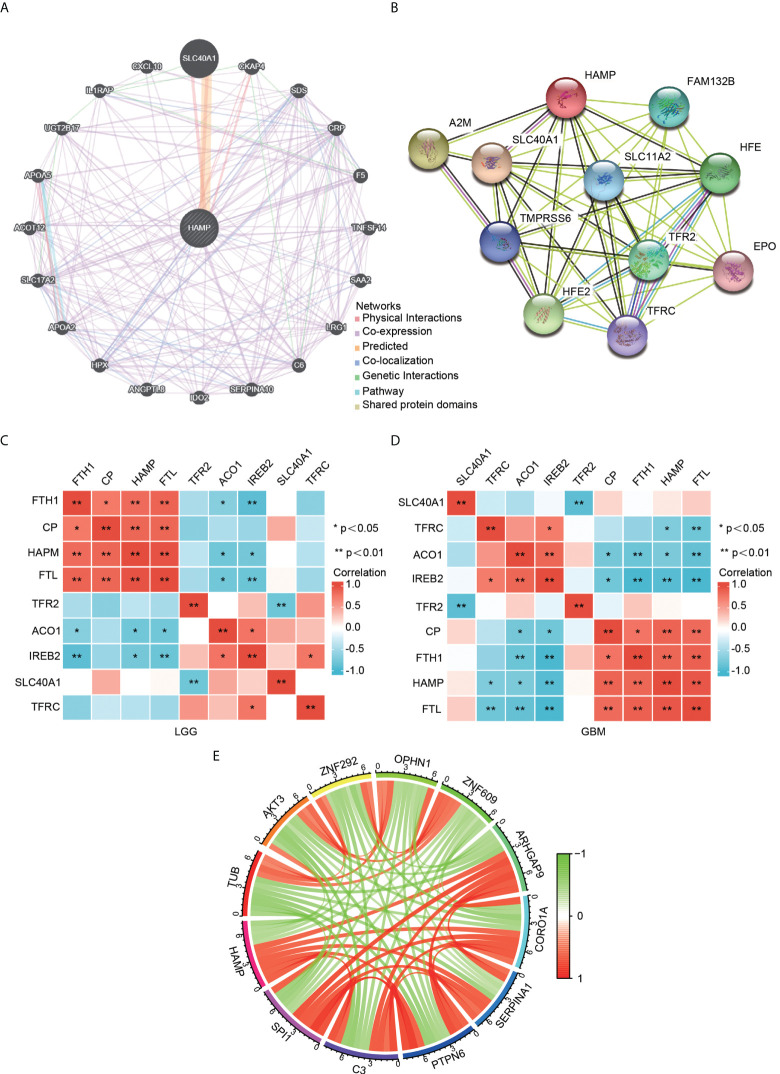
Analysis of hepcidin-interacting genes and proteins. **(A)** The gene–gene interaction network of hepcidin was constructed using GeneMANIA. **(B)** The PPI network of hepcidin was generated using STRING. **(C, D)** A heatmap shows the correlations between hepcidin and iron metabolism-related genes in LGG **(C)** and GBM **(D)**. **(E)** The gene circle map of hepcidin from the co-expression analysis. Red lines represent positive correlations with hepcidin, and green lines represent negative correlations with hepcidin. *p < 0.05 and **p < 0.01.

A gene–gene interaction network for hepcidin was constructed by using GeneMANIA. The 20 most frequently altered genes, such as SLC40A1, were closely correlated with hepcidin **(** **Figure 5A****)**. By using the STRING database, a PPI network of hepcidin was generated. There were 48 edges and 11 nodes, including SLC40A1, TFR2 and HFE **(** **Figure 5B****)**. Furthermore, the correlations between hepcidin and iron metabolism-related genes were investigated based on the TCGA database. Hepcidin was positively and significantly correlated with CP, FTH1 and FTL and negatively correlated with, ACO1 and IREB2 in LGG and GBM **(** **Figures 5C, D****)**. The first 11 genes that have a coexpression relationship with hepcidin in the CGGA database are shown by a circle diagram. The expression of hepcidin was positively correlated with the expression of SP11, C3, PTPN6, SERPINA1, CORO1A, and ARHGAP9 and was negatively correlated with the expression of TUB, AKT3, ZNF292, OPHN1 and ZNF609 **(** **Figure 5E****)**.

### Functional analysis of DEGs between the high and low hepcidin expression groups in the CGGA datasets

**Figure 6:**
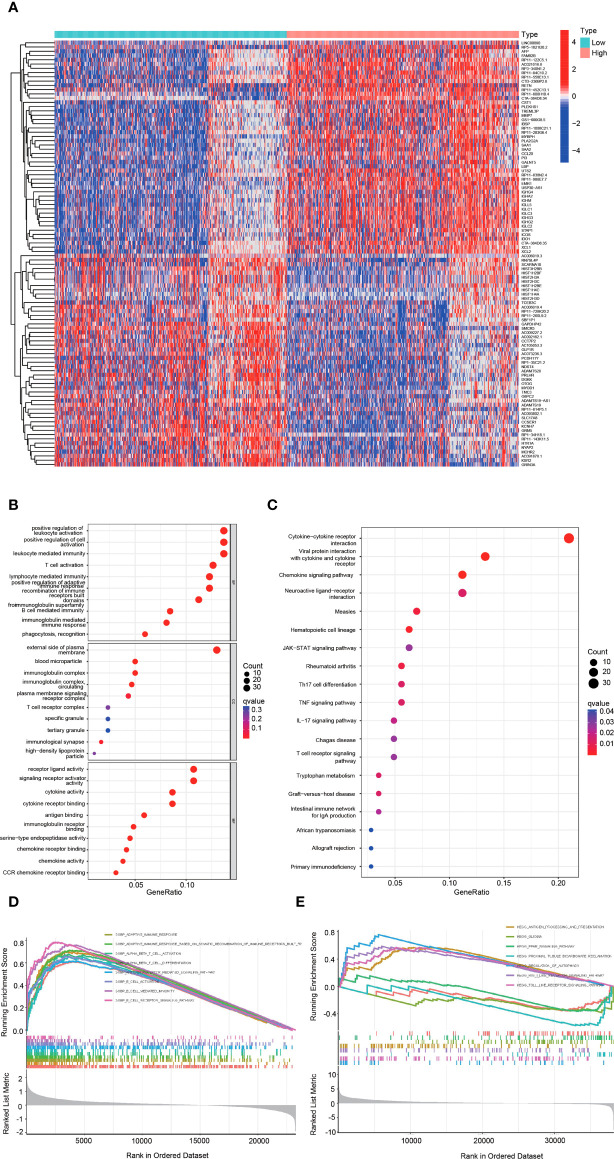
Functional analysis of DEGs between the high and low hepcidin expression groups in the CGGA dataset. **(A)** Heatmaps of the differentially expressed genes between the high and low hepcidin expression groups. **(B, C)** GO and KEGG analyses of DEGs. **(D, E)** GSEA-GO **(D)** and GSEA-KEGG **(E)** enrichment analyses of the high and low hepcidin expression groups in the CGGA dataset.

The heatmap showed the DEGs between the high and low hepcidin expression groups in the CGGA **(** **Figure 6A****)** and TCGA datasets **(** **Supplementary Figure S3A****)**. KEGG and GO enrichment analyses were used to explore hepcidin-related pathways and biological functions. The top 10 significant terms identified in biological process (BP), molecular function (MF) and cellular component (CC) enrichment analyses in CGGA **(** **Figure 6B****)** and TCGA **(** **Supplementary Figure S3B****)** datasets are presented. Notably, in terms of BPs, hepcidin was enriched in immune response-related processes, such as T cell activation and lymphocyte-, leukocyte- and B cell-mediated immunity. In addition, the meaningful KEGG pathways of the DEGs in CGGA **(** **Figure 6C****)** and TCGA **(** **Supplementary Figure S3C****)** datasets are shown. Among these pathways, many immune-related pathways were highly associated with hepcidin, including cytokine–cytokine receptor interaction, chemokine signaling pathway, T cell receptor signaling pathway, Th17 cell differentiation and so on. Furthermore, GSEA results of the CGGA **(** **Figures 6D, E****)** and TCGA **(** **Supplementary Figures S3D, E****)** datasets showed that the biological functions of the DEGs were related to the activation of T and B cells or immune-related pathways, such as antigen processing and presentation, Toll-like receptor or Rig I-like receptor signaling and glioma pathways.

### Correlation analysis between hepcidin expression and infiltrating immune cells

**Figure 7:**
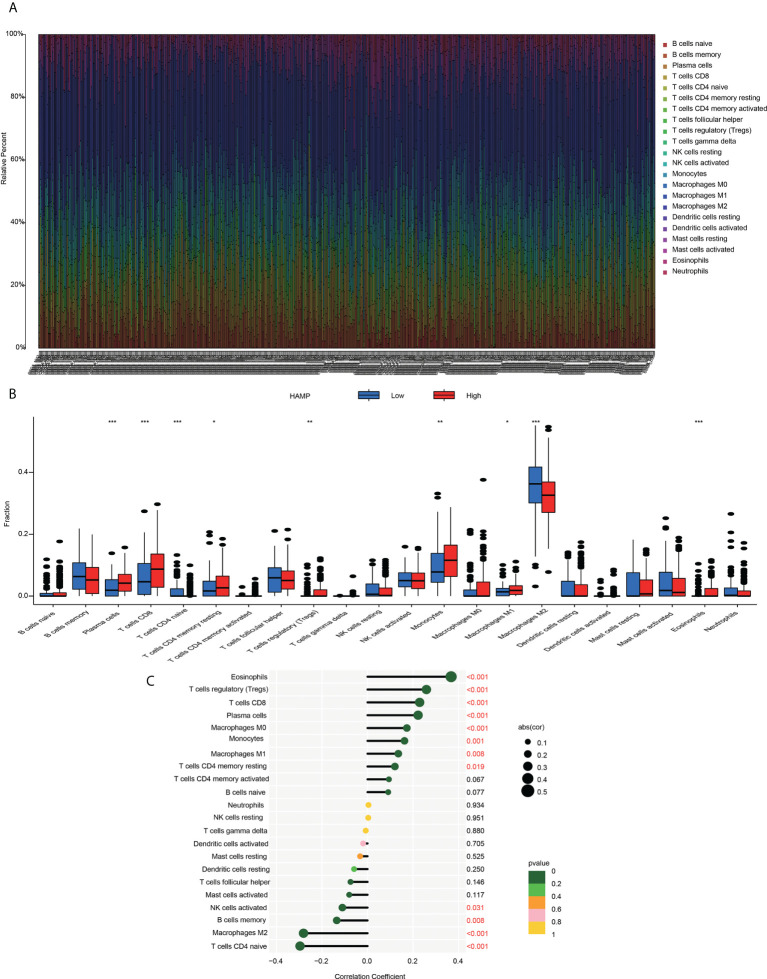
Immune cell infiltration with the CIBERSORT algorithm **(A)** The abundance ratios of immune cells in the CGGA samples. The 22 specific immune cells corresponding to one sample by different colors, as shown in the bar plot. **(B)** The varied proportions of 22 subtypes of immune cells in the high and low hepcidin groups in tumor samples. Horizontal and vertical axes represent TIICs and relative percentages, respectively. Blue and red colors represent the low and high hepcidin expression groups, respectively. **(C)** The correlation between immune infiltrating cells and hepcidin expression. The ordinate represents the name of the immune cell, and the abscissa represents the correlation coefficient. *p < 0.05, **p < 0.01, and ***p < 0.001.

**Figure 8:**
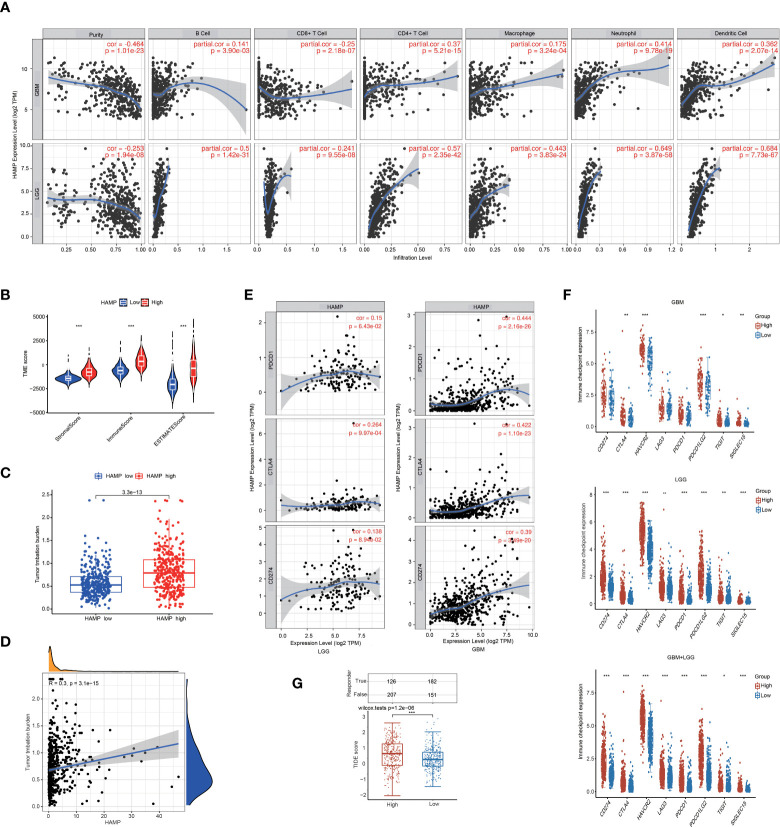
Analysis of the correlations between hepcidin expression and immune cell infiltration and immunotherapy. **(A)** hepcidin is significantly associated with tumor purity and is positively correlated with the infiltration of different immune cells using the TIMER database. **(B)** The TME scores between the high and low hepcidin expression groups in the CGGA database. **(C)** Comparison of the TMB between the high and low hepcidin expression groups in the CGGA database. **(D)** The correlation between hepcidin expression and the TMB. **(E)** Scatterplots of the correlations between hepcidin expression and PD-1, PD-L1 and CTLA-4 expression in LGG and GBM. **(F)** The effect of hepcidin on the expression of immune checkpoint genes. The expression of various immune checkpoint genes between hepcidin high-expression group and hepcidin low-expression group of patients with LGG or/and GBM was examined. **(G)** Comparison of TIDE scores between the hepcidin high-expression group and low-expression group based on TCGA database. *p < 0.05, **p < 0.01, and ***p < 0.001.

Using the established computational resource CIBERSORT, the abundance ratios of 22 immune cells in glioma samples from the CGGA **(** **Figure 7A****)** and TCGA datasets **(** **Supplementary Figure S4A****)** were explored, and the relationship between the abundance ratios of the immune cells are presented. The changes in the proportions of 22 subtypes of immune cells in the high and low hepcidin expression groups in tumor samples showed that immune cells, such as eosinophils, Tregs, CD8+ T cells, plasma cells, M1 macrophages and monocytes, were significantly positively correlated with hepcidin expression, and CD4+ naïve T cells and M2 macrophages were significantly negatively correlated with hepcidin expression in CGGA dataset (*p* < 0.05) **(** **Figures 7B, C****)**. TCGA results were basically similar to the CGGA results **(** **Supplementary Figure S4C****)**. In addition, we analyzed the correlation between hepcidin expression and six types of infiltrating immune cells, including B cells, CD4+ T cells, neutrophils, macrophages, and dendritic cells, using the TIMER database. The results showed that hepcidin expression levels had a significant positive correlation with the infiltration of B cells, CD4+ T cells, macrophages, neutrophils, and dendritic cells and negative correlations with CD8+ T cells in GBM. In LGG, these six types of cells all had positive correlations with hepcidin **(** **Figure 8A****)**.

**Figure 9:**
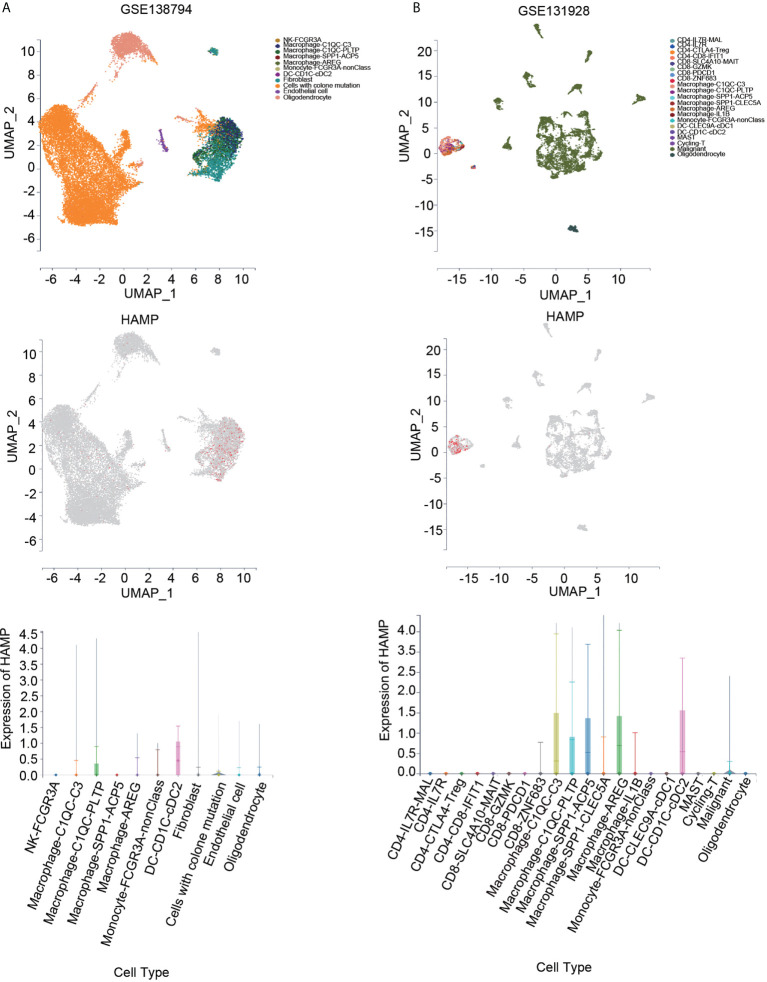
Expression of hepcidin at the single-cell level using the scTIME portal database. **(A)** UMAP plot of all cells from the original article using the GSE138794 dataset (upper panel). UMAP plot of the hepcidin gene based on scRNA-seq data (middle panel). The expression of hepcidin in different cells (lower panel). **(B)** UMAP plot of all cells from the original article using the GSE131928 dataset (upper panel). UMAP plot of the hepcidin gene based on scRNA-seq data (middle panel). The expression of hepcidin in different cells (lower panel).

We also investigated hepcidin expression in glioma samples from two single-cell RNA-seq datasets using the scTIME portal database to further confirm immune cell infiltration. Hepcidin was also expressed at high levels in macrophages according to the GSE138794 and GSE131928 datasets **(** **Figures 9A, B****)**. Additionally, hepcidin expression was also observed macrophages in the GSE84465, GSE89567, GSE131928, GSE148842 and GSE138794 datasets using the TISCH database **(** **Supplementary Figure S5****)**. Moreover, the correlations between hepcidin expression and several markers of macrophages were investigated. We observed that hepcidin expression was positively and significantly correlated with CD163, CD68, MARCO, MRC1, MSR1 and FCGR3A expression in LGG and GBM **(** **Supplementary Figures S6A, B****)**.

### Correlation between hepcidin expression and various immune characteristics

To deepen our understanding of hepcidin crosstalk with the immune response, we validated the correlations between hepcidin expression and diverse immune signatures in both LGG and GBM using the TIMER database. The genes listed in **Table 1** were used to characterize immune cells. Tumor purity is an important aspect affecting the analysis of immune infiltration in clinical cancer biopsies. After adjusting for tumor purity, hepcidin expression was significantly associated with most immune markers in diverse types of immune cells in LGG and GBM **(** **Table 1****)**.

**Table 1:** Correlation analysis between hepcidin and gene markers of immune cells in LGG and GBM in TIMER.

Description	Gene markers	LGG				GBM			
		None		Purity		None		Purity	
		Cor	*p*	Cor	*p*	Cor	*p*	Cor	*p*
**B cell**	CD19	0.345	***	0.313	***	0.225	**	0.18	*
	CD79A	0.28	***	0.311	***	0.278	***	0.289	***
**T cell (general)**	CD3D	0.48	***	0.46	***	0.414	***	0.218	*
	CD3E	0.487	***	0.474	***	0.321	***	0.143	0.0948
	CD2	0.507	***	0.492	***	0.383	***	0.188	*
**CD8+ T cell**	CD8A	0.372	***	0.31	***	0.13	0.109	-0.003	0.976
	CD8B	0.221	***	0.182	***	0.248	**	0.129	0.134
**Monocyte**	CD86	0.62	***	0.597	***	0.537	***	0.335	***
	CSF1R	0.544	***	0.51	***	0.553	***	0.376	***
**TAM**	CCL2	0.545	***	0.295	***	0.667	***	0.56	***
	CD68	0.545	***	0.405	***	0.56	***	0.374	***
	IL10	0.561	***	0.392	***	0.685	***	0.548	***
**M1**	IRF5	0.578	***	0.557	**	0.453	***	0.253	**
	PTGS2	0.214	***	0.161	***	0.475	***	0.384	***
	NOS2	-0.099	*	-0.113	*	-0.144	0.0749	-0.103	0.233
**M2**	CD163	0.331	***	0.32	***	0.587	***	0.448	***
	VSIG4	0.448	***	0.405	***	0.66	***	0.52	***
	MS4A4A	0.375	***	0.374	***	0.657	***	0.527	***
**Neutrophil**	CEACAM8	-0.063	0.156	-0.064	0.164	-0.096	0.239	-0.167	0.05132
	ITGAM	0.61	***	0.584	***	0.536	***	0.37	***
	CCR7	0.296	***	0.291	***	0.38	***	0.261	**
**Natural killer cell**	KIR2DL1	0.05	0.256	0.073	0.113	0.236	**	0.195	*
	KIR2DL3	0.174	***	0.187	***	0.035	0.669	-0.029	0.739
	KIR2DL4	0.409	***	0.418	***	0.262	**	0.19	*
	KIR3DL1	0.012	0.782	0.017	0.704	0.041	0.618	-0.024	0.779
	KIR3DL2	0.185	***	0.18	***	0.12	0.14	0.1	0.247
	KIR3DL3	-0.021	0.632	-0.017	0.704	0.034	0.675	0.077	0.368
	KIR2DS4	0.149	***	0.144	***	0.191	*	0.096	0.265
**Dendritic cell**	HLA-DPB1	0.687	***	0.676	***	0.494	***	0.331	***
	HLAD-QB1	0.549	***	0.532	***	0.328	***	0.197	*
	HLA-DRA	0.715	***	0.7	***	0.574	***	0.402	***
	HLA-DPA1	0.675	***	0.663	***	0.432	***	0.3	***
	CD1C	0.278	***	0.274	***	0.302	***	0.111	0.197
	NRP1	0.208	***	0.234	***	0.353	***	0.294	***
	ITGAX	0.469	***	0.43	***	0.26	**	0.107	0.211

*p < 0.05, **p < 0.01, and ***p <0.001.

We also examined the correlation between hepcidin expression and various functional T cells, including Th1 cells, Th1-like cells, Th2 cells, Tregs, resting Tregs, effector Tregs, effector T cells, naïve T cells, effector memory T cells, resistant memory T cells, and exhausted T cells **(** **Table 2****)**. By using the TIMER database, we found that the hepcidin expression level was significantly correlated with 34 of 38 T cell markers in LGG and with 23 of 38 T cell markers in GBM after adjusting for tumor purity **(** **Table 2****)**.

**Table 2:** Correlation analysis between hepcidin and gene markers of different types of T cells in LGG and GBM in TIMER.

Description	Gene markers	LGG				GBM			
		None		Purity		None		Purity	
		Cor	*p*	Cor	*p*	Cor	*p*	Cor	*p*
Th1	TBX21	0.227	***	0.283	***	0.016	0.847	0.08	0.351
	STAT4	0.054	0.217	0.009	0.839	0.212	**	0.002	0.981
	STAT1	0.442	***	0.434	***	-0.068	0.402	-0.017	0.846
	TNF	0.251	***	0.205	***	0.384	***	0.243	**
	IFNG	0.238	***	0.219	***	0.075	0.358	0.037	0.665
Th1-like	HAVCR2	0.657	***	0.637	***	0.6	***	0.416	***
	IFNG	0.238	***	0.219	***	0.075	0.358	0.037	0.665
	CXCR3	0.498	***	0.48	***	0.328	***	0.193	*
	BHLHE40	0.285	***	0.214	***	0.333	***	0.281	***
	CD4	0.573	***	0.557	***	0.595	***	0.421	***
Th2	STAT6	0.492	***	0.447	***	0.358	***	0.185	*
	STAT5A	0.558	***	0.518	***	0.226	**	0.118	0.168
Treg	FOXP3	-0.167	***	-0.149	**	0.098	0.226	0.03	0.727
	CCR8	0.123	**	0.135	**	0.299	***	0.2	*
	TGFB1	0.515	***	0.483	***	0.405	***	0.27	**
Resting Treg	FOXP3	-0.167	***	-0.149	**	0.098	0.226	0.03	0.727
	IL2RA	0.236	***	0.255	***	0.568	***	0.465	***
Effector Treg	FOXP3	-0.167	***	-0.149	**	0.098	0.226	0.03	0.727
	CCR8	0.123	**	0.135	**	0299	***	0.2	*
	TNFRSF9	0.134	**	0.127	**	0.431	***	0.341	***
Effector T cell	CX3CR1	0.521	***	0.486	***	0.354	***	0.271	**
	FGFBP2	0.365	***	0.329	**	0.103	0.204	0.007	0.938
	FCGR3A	0.731	***	0.714	***	0.709	***	0.585	***
Naïve T cell	CCR7	0.296	***	0.291	***	0.38	***	0.261	**
	SELL	-0.142	**	-0.169	***	0.423	***	0.289	***
Effector memory T cell	DUSP4	-0.107	*	-0.087	0.0563	-0.025	0.755	0.01	0.91
	GZMK	0.484	***	0.476	***	0.321	***	0.155	0.0699
	GZMA	0.482	***	0.475	***	0.414	***	0.271	**
Resident memory T cell	CD69	0.666	***	0.639	***	0.434	***	0.277	**
	CXCR6	0.454	***	0.427	***	0.362	***	0.174	*
	MYADM	0.243	**	0.155	***	0.25	**	0.208	*
General memory T cell	CCR7	0.296	***	0.291	***	0.38	***	0.261	**
	SELL	-0.142	**	-0.169	***	0.423	***	0.289	***
	IL7R	0.339	***	0.293	0.0181	0.404	***	0.252	**
Exhausted T cell	HAVCR2	0.657	***	0.637	***	0.6	***	0.416	***
	LAG3	0.176	***	0.211	***	-0.13	0.109	-0.087	0.314
	CXCL13	-0.115	**	-0.07	0.125	0.305	***	0.247	**
	LAYN	-0.12	**	-0.124	**	0.341	***	0.16	0.0611

*p < 0.05, **p < 0.01, and ***p < 0.001.

To evaluate the proportions of immune and stromal components in the TME, the immune and stromal scores of each glioma sample from the CGGA were calculated using the “limma” and “estimate” packages in R language. A violin plot of the TME score showed that the low hepcidin expression group had lower TME scores than the high hepcidin expression group **(** **Figure 8B****)**. In the TCGA glioma database, the TMB of the high hepcidin expression group was significantly higher than that of the low hepcidin expression group, and there was a significant positive correlation between hepcidin expression and TMB **(** **Figures 8C, D****)**. We further investigated the interrelationship between hepcidin expression and well-known T cell checkpoints, such as PD-1, PD-L1 and CTLA-4, in the GEPIA database. The expression of hepcidin was significantly correlated with the expression of PD-1, PD-L1 and CTLA-4 in LGG and GBM **(** **Figure 8E****)**. Furthermore, patients with LGG and/or GBM were divided into hepcidin high-expression and low-expression groups. As shown in **Figure 8F**, the expression of most immune checkpoint genes, including PD-1, PD-L1, PDCD1LG2, CTLA4, HAVCR2, LAG3, TIGHT and SIGLEC15, was obviously higher in the hepcidin high-expression group than in the hepcidin low-expression group of patients with LGG and GBM. In addition, the effect of hepcidin expression on predicting the immunotherapy response was further evaluated in TCGA cohort. The hepcidin high-expression group had a higher TIDE score **(** **Figure 8G****)**. Patients with high TIDE scores tended to respond poorly to immunotherapy, suggesting that immunotherapy is unlikely to benefit patients with high hepcidin expression. Together, these findings suggest that hepcidin expression is significantly related to immune cell infiltration and that hepcidin plays an important role in immune escape in the brain cancer microenvironment.

## Discussion

The treatment of brain tumor faces unique and severe challenges due to the existence of the blood-brain barrier (BBB) (29, 30). The BBB is a highly selective semipermeable barrier that separates the blood from the brain, but also impediments drug penetration. The initial treatment for brain tumors is surgery (if feasible and safe), and maximal resection is strongly correlated with longer OS (31). Patients usually receive radiation and chemotherapy as an adjunct. Radiotherapy with a total dose of 60 Gy, as primary or postoperative treatment, improved OS and progression-free survival (PFS) (32). Concomitant administration of the oral alkylating agent temozolomide significantly increased OS in newly diagnosed GBM patients (33). However, although radiotherapy and temozolomide improve survival, tumor progression and recurrence usually occur due to the development of temozolomide resistance. In recent years, new therapies such as molecular targeted therapy (34), alternating electric field therapy (35), ultrasound focusing (36) and nanotherapy (37) have achieved great progress, but the prognosis of patients with glioma has not improved substantially. This lack of improvement may be due to the fact that the complex pathological process of glioma has not been fully elucidated and effective biomarkers for an accurate diagnosis and molecular targeted therapy are lacking. However, with the development of new immunotherapy aimed at reviving the anti-tumor immune response, the prognosis of patients with various advanced hematological diseases and solid malignant tumors has significantly improved (38–40). Immune checkpoint inhibitors, such as anti-PD-1 and anti-CTLA4 therapy, enhance T cell activity and inhibit immunosuppression in the TME (41). The presence of some biomarkers, such as mutant IDH, O6-methylguanine DNA methyltransferase (MGMT) promoter methylation, epidermal growth factor receptor amplification and p53 mutation, have predictive and diagnostic potential in gliomas (42). Therefore, we need to find more effective treatments to improve the survival rate of these patients. Some genes and transcription factors have been shown to play a key role in the occurrence, development and evolution of gliomas. Improving the understanding of the pathogenesis of glioma and the recognition of key molecular markers will help to improve the diagnostic accuracy of glioma and identify new therapeutic targets to achieve better clinical results (43, 44).

At present, there is no study on the expression of hepcidin in gliomas. In the present study, we showed higher expression of hepcidin in glioma than in normal brain tissues, and hepcidin expression was significantly upregulated with increasing glioma grade in the CGGA, TCGA, Rembrandt and Gravendeel glioma databases **(** **Figure 1****)**. Subsequently, the Kaplan–Meier curve and statistical tests revealed a significantly lower OS rate for patients with glioma presenting high hepcidin expression than that of patients with low hepcidin expression in the four glioma datasets **(** **Figure 2****)**. In addition, the clinical prognostic significance of hepcidin in glioma patients was investigated. High hepcidin expression was significantly correlated with the tumor grade, age, PRS type, IDH mutation, chemotherapy status, and 1p19q codeletion status in patients with glioma from CGGA datasets **(** **Figure 3****)**. Through the univariate and multivariate independent prognostic analyses of CGGA and TCGA datasets, we found that hepcidin is useful as an independent prognostic factor for glioma. At the same time, we constructed a prognostic model based on the parameters of the multivariate model, and found that the model was stable in predicting the prognosis **(** **Figure 4** **and** **Supplementary Figure S2****)**. Additionally, we found that hepcidin expression was negatively correlated with temozolomide sensitivity, indicating that the higher the expression of hepdicin, the lower the IC50 value of temozolomide in brain tumors based on the GDSC database using the R package pRRophetic **(** **Supplementary Figures S7A, B****)**. These results suggested that hepdicin may affected the sensitivity of patients with GBM and LGG to temozolomide treatment **(** **Supplementary Figures S7A, B****)**. Together, hepcidin may be an independent prognostic biomarker in glioma and may facilitate the development of targeted precision oncology.

By using the GeneMANIA and STRING databases, hepcidin-interacting genes and proteins were identified. The results suggested that iron metabolism-related genes were closely correlated with hepcidin expression. The function of hepcidin in glioma remains unclear, but one hypothesis is that it is associated with local iron homeostasis. Furthermore, the correlations between hepcidin and iron metabolism-related genes were investigated based on the TCGA database. Hepcidin was negatively and significantly correlated with CP, FTH1 and FTL and negatively correlated with ACO1 and IREB2 in LGG and GBM **(** **Figure 5****)**. Human hepcidin is highly expressed in hepatocytes. In the central nervous system, iron enters the brain mainly through Tf receptor 1 (TFRC) and iron transporters across the BBB (45, 46). First, the circulating iron-Tf complex binds to TfR1 expressed on the endothelial surface of the BBB to form a Fe^3+^-Tf-TfR1 complex. Then, after internalization and acidification, iron is released in the form of Fe^2+^, and Fe^2+^ is transported to the lysosomal membrane by divalent metal ion transporter 1 (DMT1). If the iron in the cell cannot be used immediately, it is usually stored in the form of ferritin in the form of Fe^3+^, which the body releases from ferritin or during lysosome degradation as needed. In addition, iron can enter the mitochondrial matrix through mitochondrial ferritin. Only a small portion of iron can be used as a variable iron pool in the ongoing biochemical process (45). The distribution of iron in the brain is mainly concentrated in the pars compacta of the substantia nigra, periventricular organs, globus pallidus and oligodendrocytes, and its distribution is uneven in different parts and different brain cells. Glial cells have the highest iron content, in which almost all Tf and different types of iron metabolism-related proteins are expressed in the brain (46). Therefore, glial cells are indispensable for maintaining iron homeostasis and normal physiological function in the brain. An increasing number of studies have shown that an increase in serum hepcidin is associated with a variety of cancers (47). In addition, recent studies have shown that cancer cells produce hepcidin. In breast cancer (48), prostate cancer (7, 47), pancreatic cancer (49), lung cancer (21) and thyroid cancer (50), hepcidin expression is significantly increased. More importantly, the downregulation of tumor hepcidin expression can also strongly inhibit tumor growth (51). In addition, some studies have shown that the increased expression of hepcidin may increase the chemotherapy resistance of tumors (48). It is speculated that this mechanism may be related to IL-6 (52, 53). Hepcidin has been considered a particularly attractive target, and agents that inhibit hepcidin are under active investigation as potential therapies for cancer treatment. However, it should be noted that too little hepcidin will cause iron deposition, which can also lead to the formation and occurrence of tumors. Therefore, the ways and methods of reducing hepcidin expression still need to be further explored.

To further clarify the mechanism of hepcidin in the occurrence and development of gliomas, KEGG and GO enrichment analyses were used to explore the hepcidin-related pathways and biological functions. Hepcidin was closely correlated with immunity **(** **Figure 6** and **Supplementary Figure S3****)**. Based on accumulating evidence, TME plays an important role in the growth and invasion of gliomas (42, 54, 55). The TME is a complex dynamic evolutionary environment that mainly includes a variety of immune cells, stromal cells and cytokines released by cells. Cytokines and chemokines produced locally by tumors and their interactions with extracellular matrix components reprogram infiltrating immune cells to obtain different functional phenotypes, which guide the immune system into inflammatory or anti-inflammatory responses and then reshape the surrounding microenvironment and promote tumor proliferation and progression. At present, an increasing amount of data shows that iron metabolism in the TME is also an important factor in maintaining the survival of cancer cells. Cancer cells have unique characteristics of iron metabolism, which provide the necessary iron for the rapid proliferation and metastasis of cancer cells. Compared with malignant tumor cells, the genetic stability of normal cells in the TME determines the therapeutic stability of this target (56). As a new treatment mode, immunotherapy has attracted increasing attention from researchers (57). Various preclinical studies have shown that immunotherapy-based methods have been successful in animal models. A large number of phase I and II clinical trials have shown that immunotherapy is safe and, in some cases, prolongs OS and PFS (36, 58, 59). In gliomas, the therapeutic effect of ICB has always been unpredictable and uncommon, and only 8% of GBM patients show a clear response (60, 61). Therefore, understanding the factors and factors affecting the TME is of great significance to the treatment of gliomas. Here, using the CIBERSORT algorithm and TIMER web portal, we first report that high hepcidin expression in glioma correlated with the increased infiltration of B cells, CD4+ T cells, CD8+ T cells, neutrophils, macrophages, and dendritic cells (**Figures 7** and **Supplementary Figure S4**). Single-cell RNA sequencing analysis further suggested that hepcidin was expressed at high levels in macrophages. Moreover, a significant association between hepcidin and various immune cell marker sets was observed in glioma **(** **Table 1**, **Table 2****)**. Hepcidin expression was also positively correlated with PD-1 and CTLA-4 expression. More importantly, we found that there was a significant positive correlation between hepcidin and TMB **(** **Figure 8****)**. Furthermore, most immune checkpoint genes, including PD-1, PD-L1, PDCD1LG2, CTLA4, HAVCR2, LAG3, TIGHT and SIGLEC15, were highly expressed in the hepcidin high-expression group of patients with LGG and GBM **(** **Figure 8F****)**. TIDE was further used to predict immunotherapy responses of different hepcidin expression groups and to verify this conclusion. TIDE scores were lower in the hepcidin low-expression group than in the hepcidin high-expression group, implying that immunotherapy may provide a greater benefit to patients in the hepcidin low-expression group **(** **Figure 8G****)**. Our study identified important roles for and the prognostic potential of hepcidin, a key factor involved in iron metabolism, in glioma and its relationship with immunity, which will provide a new idea for the clinical treatment of glioma.

However, our research still has many limitations. For example, this research is mainly focused on the analysis of databases. The roles of hepcidin must be validated in a larger patient population. Second, we mainly analyzed the prognostic value of hepcidin expression in glioma, the signaling pathways involved and the relationship with immune cell infiltration using bioinformatics methods. Additional *in vivo* and *in vitro* experiments are required to verify the function of hepcidin. Third, the specific mechanisms of the upstream and downstream pathways of hepcidin remain to be further explored.

In summary, these findings suggest that hepcidin could be a new target for immune-related glioma therapy. However, the exact role of hepcidin in the tumor immune microenvironment still needs to be further explored.

## Data availability statement

The original contributions presented in the study are included in the article/**Supplementary Material**. Further inquiries can be directed to the corresponding authors.

## Ethics statement

The studies involving human participants were reviewed and approved by the Ethics Committee of the Second Hospital of Hebei Medical University approved this study (Approval #: 2017-P035). The patients/participants provided their written informed consent to participate in this study.

## Author contributions

Study concept and design: YC, KT, and TD. Acquisition of data: TD, BZ, and KT. Analysis and interpretation of data: TD, BZ, KT, FW, and NH. Statistical analysis: RZ, CW, and XL. Drafting of the manuscript: TD and KT. Critical revision and final approval of the manuscript: KT and YC. Study supervision: KT and YC. Obtained funding: YC. All authors contributed to the article and approved the submitted version.

## Funding

This research was supported by the National Natural Science Foundation of China (31471035), Medical Science Research Project of Health Commission of Hebei Province (20210970) and Geriatric Disease Prevention Project of Hebei Province (303-2022-46-02).

## Conflict of interest

The authors declare that the research was conducted in the absence of any commercial or financial relationships that could be construed as a potential conflict of interest.

## Publisher’s note

All claims expressed in this article are solely those of the authors and do not necessarily represent those of their affiliated organizations, or those of the publisher, the editors and the reviewers. Any product that may be evaluated in this article, or claim that may be made by its manufacturer, is not guaranteed or endorsed by the publisher.

## Supplementary material

Supplementary Figure 1 Analysis of the relationship between hepcidin expression and clinical features of patients in TCGA dataset. **(A)**The relationship between hepcidin expression and clinical features of patients in TCGA dataset. **(B)** Relationships between hepcidin expression and different clinical features. ****p* < 0.001.

Supplementary Figure 2 Cox regression analysis and establishment of the prognostic model using TCGA dataset. **(A)** The univariate analysis of hepcidin expression in TCGA dataset. **(B)** The multivariate analysis of hepcidin expression in TCGA dataset. **(C)** The nomogram was constructed based on four factors for predicting 1-year, 3-year or 5-year survival of patients with glioma in TCGA. **(D)** The calibration plots of internal validation in TCGA dataset showed good consistency in predicting 1-year, 3-year or 5-year survival. **(E)** The 1-year, 3-year and 5-year ROC curves for TCGA dataset. **p < 0.01 and ***p < 0.001.

Supplementary Figure 3 Functional analysis of DEGs between high and low hepcidin expression groups using TCGA dataset. **(A)** Heatmaps of the DEGs between hepcidin high and low expression groups. **(B, C)** The GO and KEGG analyses of DEGs. **(D, E)** GSEA of GO **(D)** and KEGG **(E)** analyses of hepcidin high and low expression groups in TCGA dataset.

Supplementary Figure 4 Analysis of immune cell infiltration with the CIBERSORT algorithm in TCGA database. **(A)** The abundance ratios of immune cells in TCGA samples. The 22 specific immune cells that corresponded to one sample are indicated with different colors in the bar plot. **(B)** The correlation analysis of cells in TCGA database. **(C)** The changes in the proportions of 22 subtypes of immune cells in high and low hepcidin expression groups of tumor samples. Horizontal and vertical axes represent TIICs and relative percentages, respectively. Blue and red colors represent low and high hepcidin expression groups, respectively.

Supplementary Figure 5 Hepcidin expression in different cells was analyzed in GSE84465, GSE89567, GSE131928, GSE148842 and GSE138794 datasets using the TISCH database.

Supplementary Figure 6 The correlations between hepcidin and expression of macrophage markers based on the TCGA database. **(A)** Scatterplots of the correlations between hepcidin expression and CD163, CD68, MARCO, MRC1, MSR1 and FCGR3A expression in GBM. **(B)** Scatterplots of the correlations between hepcidin expression and CD163, CD68, MARCO, MRC1, MSR1 and FCGR3A expression in LGG.

Supplementary Figure 7 The effect of hepcidin expression on temozolomide sensitivity. **(A)** The higher the expression of hepdicin, the lower the IC50 value of temozolomide in brain tumors. **(B)** Hepcidin expression was negatively correlated with temozolomide sensitivity in brain tumors. ****p < 0.0001.
