# Erratum: Hepcidin is upregulated and is a potential therapeutic target associated with immunity in glioma

**DOI:** 10.3389/fonc.2022.1085757

**Published:** 2022-12-06

**Authors:** Frontiers Production Office

**Affiliations:** Frontiers Media SA, Lausanne, Switzerland

**Keywords:** hepcidin, glioma, prognostic biomarker, immune cell infiltration, iron

Due to a production error, there was an error in affiliation 1. Instead of “Key Laboratory of Molecular and Cellular Biology of Ministry of Education, Key Laboratory of Animal Physiology, Biochemistry and Molecular Biology of Hebei Province, College of Life Sciences, Shijiazhuang, China”, it should be “Key Laboratory of Molecular and Cellular Biology of Ministry of Education, Key Laboratory of Animal Physiology, Biochemistry and Molecular Biology of Hebei Province, College of Life Sciences, Hebei Normal University, Shijiazhuang, China”.

The publisher apologizes for this mistake. The original version of this article has been updated.

