# Supplementary figures and images for: Hepcidin is upregulated and is a potential therapeutic target associated with immunity in glioma
Source: Front Oncol. 2022 Sep 27;12:963096. doi: 10.3389/fonc.2022.963096 (PMC9552819; doi:10.3389/fonc.2022.963096)

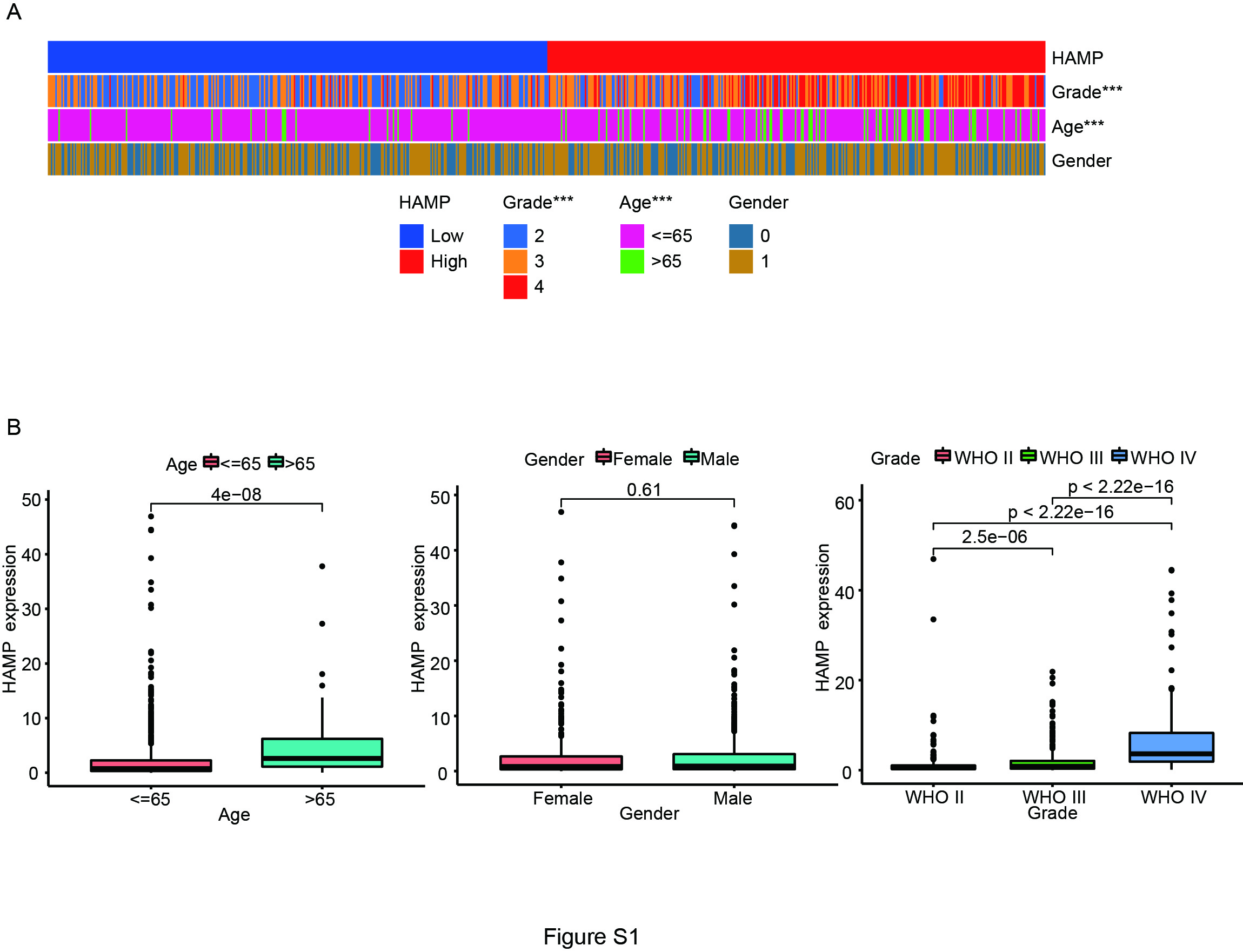

Supplement: Supplementary Figure 1 — Analysis of the relationship between hepcidin expression and clinical features of patients in TCGA dataset. (A)The relationship between hepcidin expression and clinical features of patients in TCGA dataset. (B) Relationships between hepcidin expression and different clinical features. ***p < 0.001. [file Image_1.jpeg]

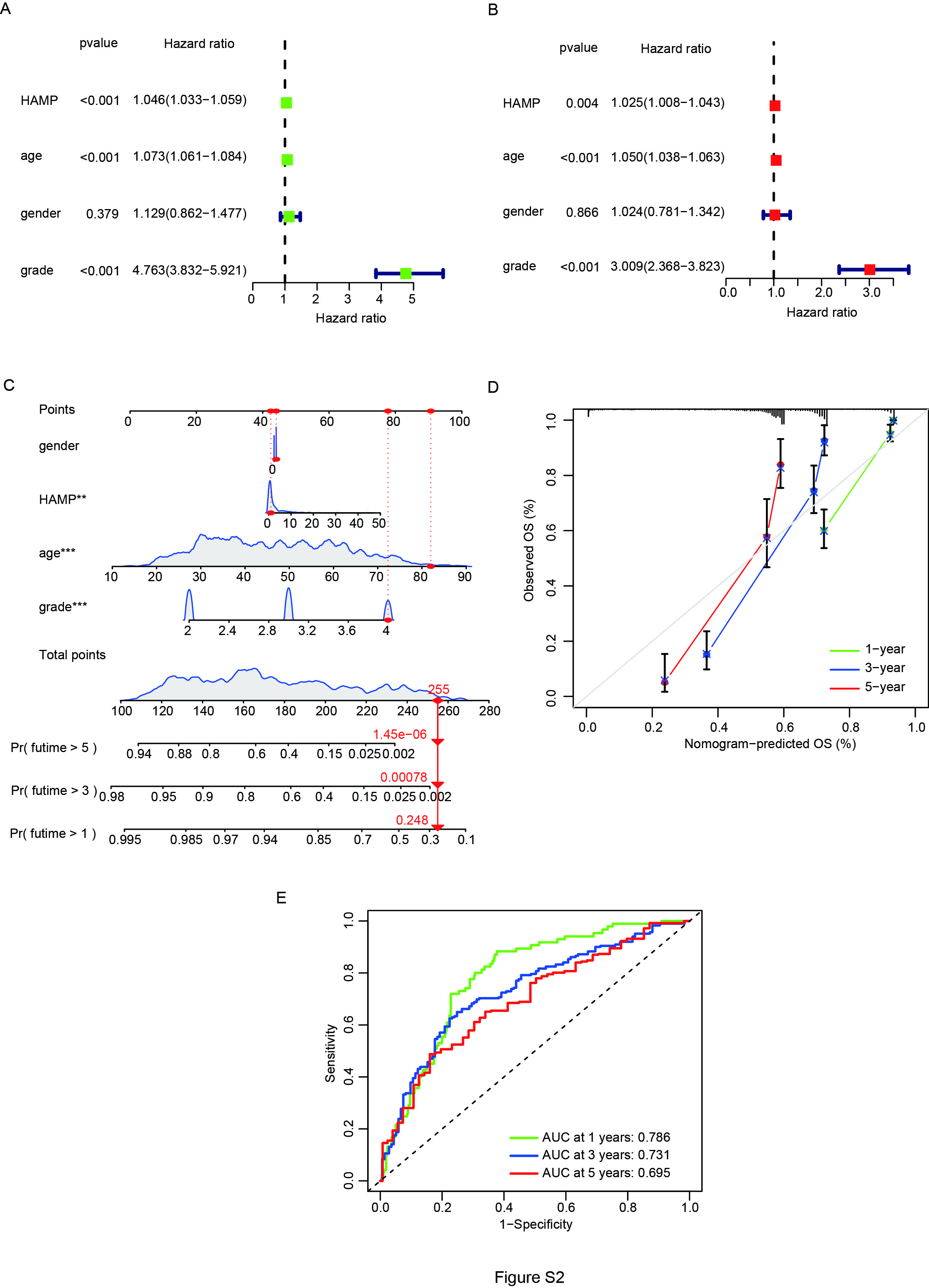

Supplement: Supplementary Figure 2 — Cox regression analysis and establishment of the prognostic model using TCGA dataset. (A) The univariate analysis of hepcidin expression in TCGA dataset. (B) The multivariate analysis of hepcidin expression in TCGA dataset. (C) The nomogram was constructed based on four factors for predicting 1-year, 3-year or 5-year survival of patients with glioma in TCGA. (D) The calibration plots of internal validation in TCGA dataset showed good consistency in predicting 1-year, 3-year or 5-year survival. (E) The 1-year, 3-year and 5-year ROC curves for TCGA dataset. **p < 0.01 and ***p < 0.001. [file Image_2.jpeg]

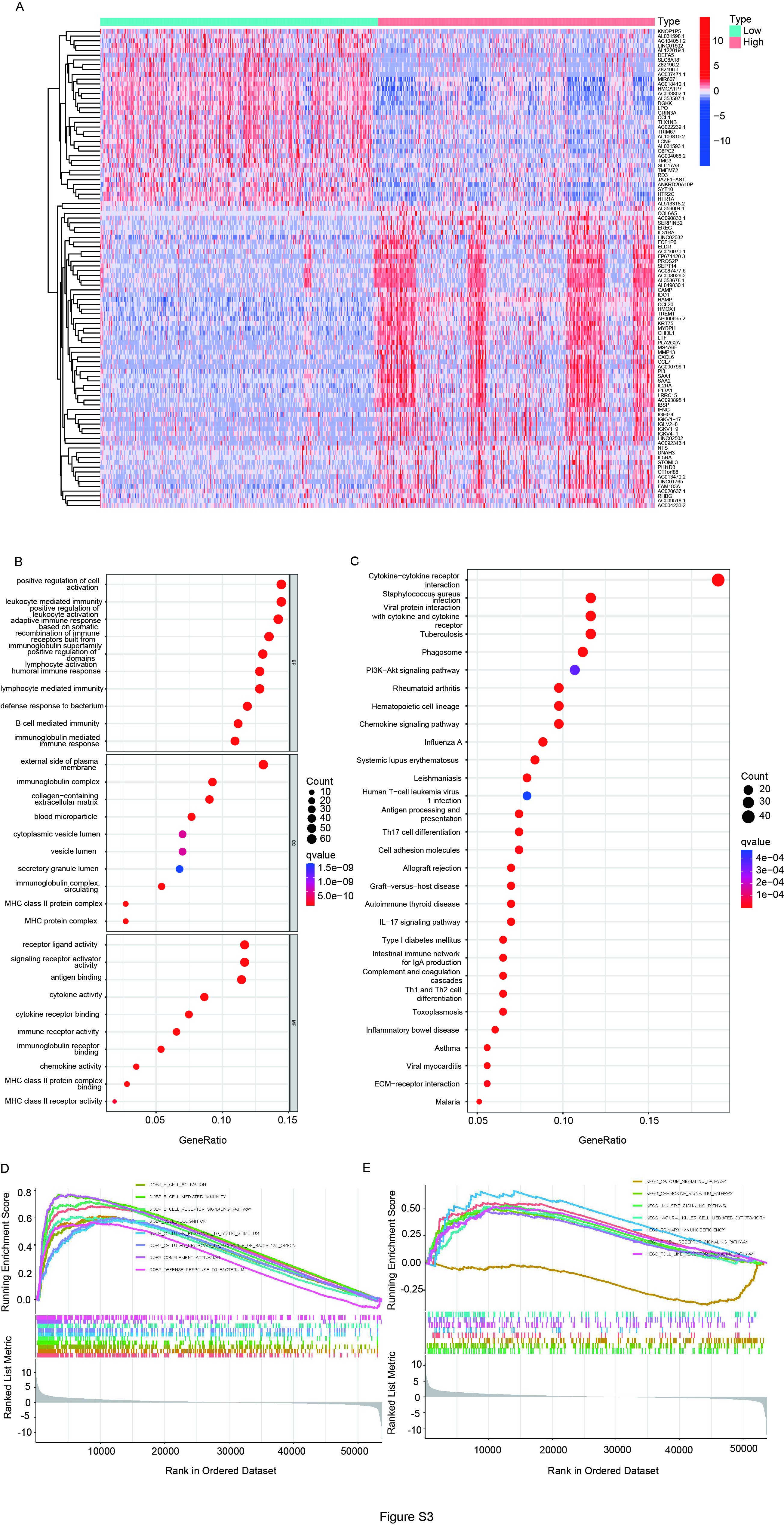

Supplement: Supplementary Figure 3 — Functional analysis of DEGs between high and low hepcidin expression groups using TCGA dataset. (A) Heatmaps of the DEGs between hepcidin high and low expression groups. (B, C) The GO and KEGG analyses of DEGs. (D, E) GSEA of GO (D) and KEGG (E) analyses of hepcidin high and low expression groups in TCGA dataset. [file Image_3.jpeg]

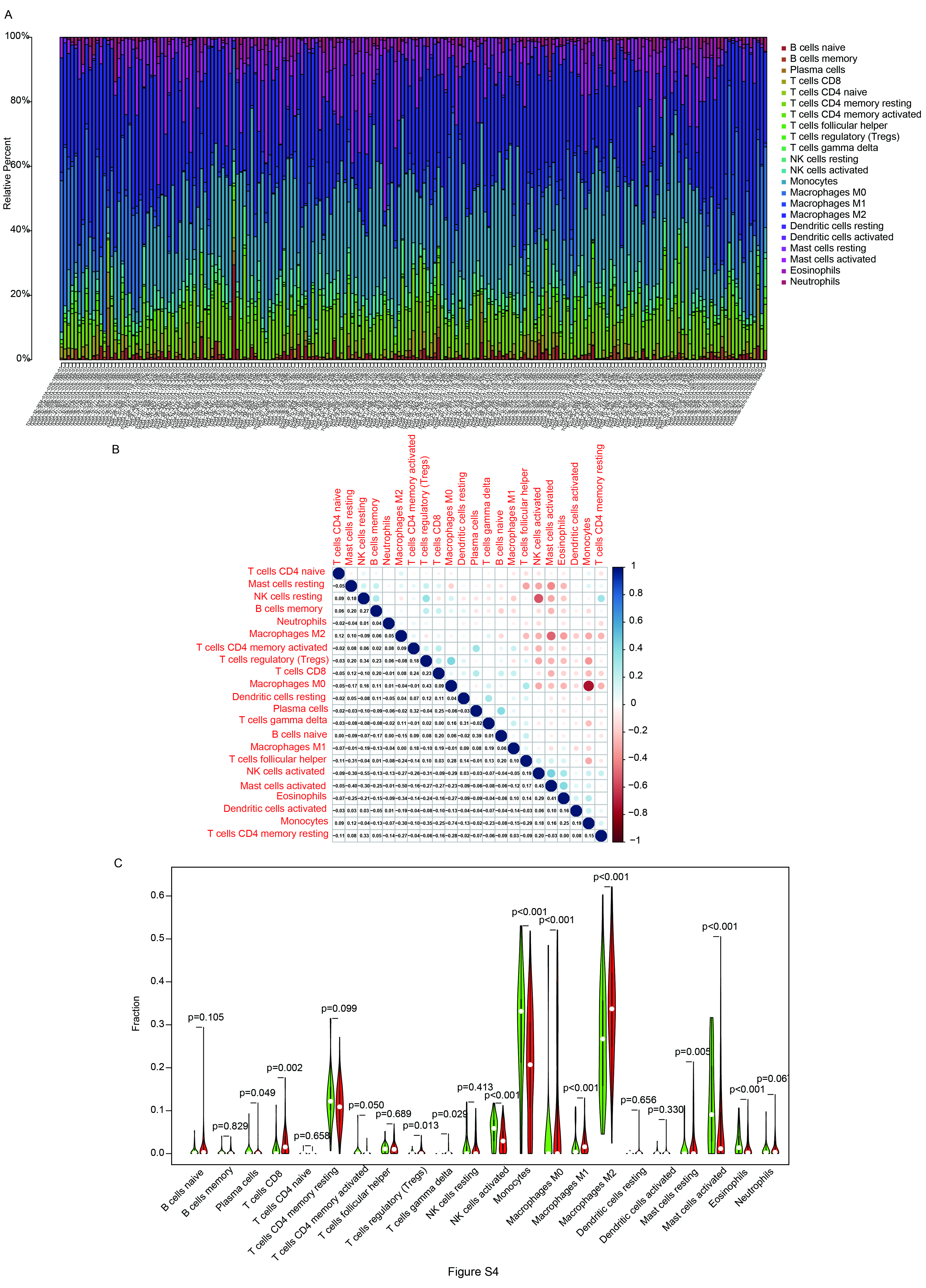

Supplement: Supplementary Figure 4 — Analysis of immune cell infiltration with the CIBERSORT algorithm in TCGA database. (A) The abundance ratios of immune cells in TCGA samples. The 22 specific immune cells that corresponded to one sample are indicated with different colors in the bar plot. (B) The correlation analysis of cells in TCGA database. (C) The changes in the proportions of 22 subtypes of immune cells in high and low hepcidin expression groups of tumor samples. Horizontal and vertical axes represent TIICs and relative percentages, respectively. Blue and red colors represent low and high hepcidin expression groups, respectively. [file Image_4.jpeg]

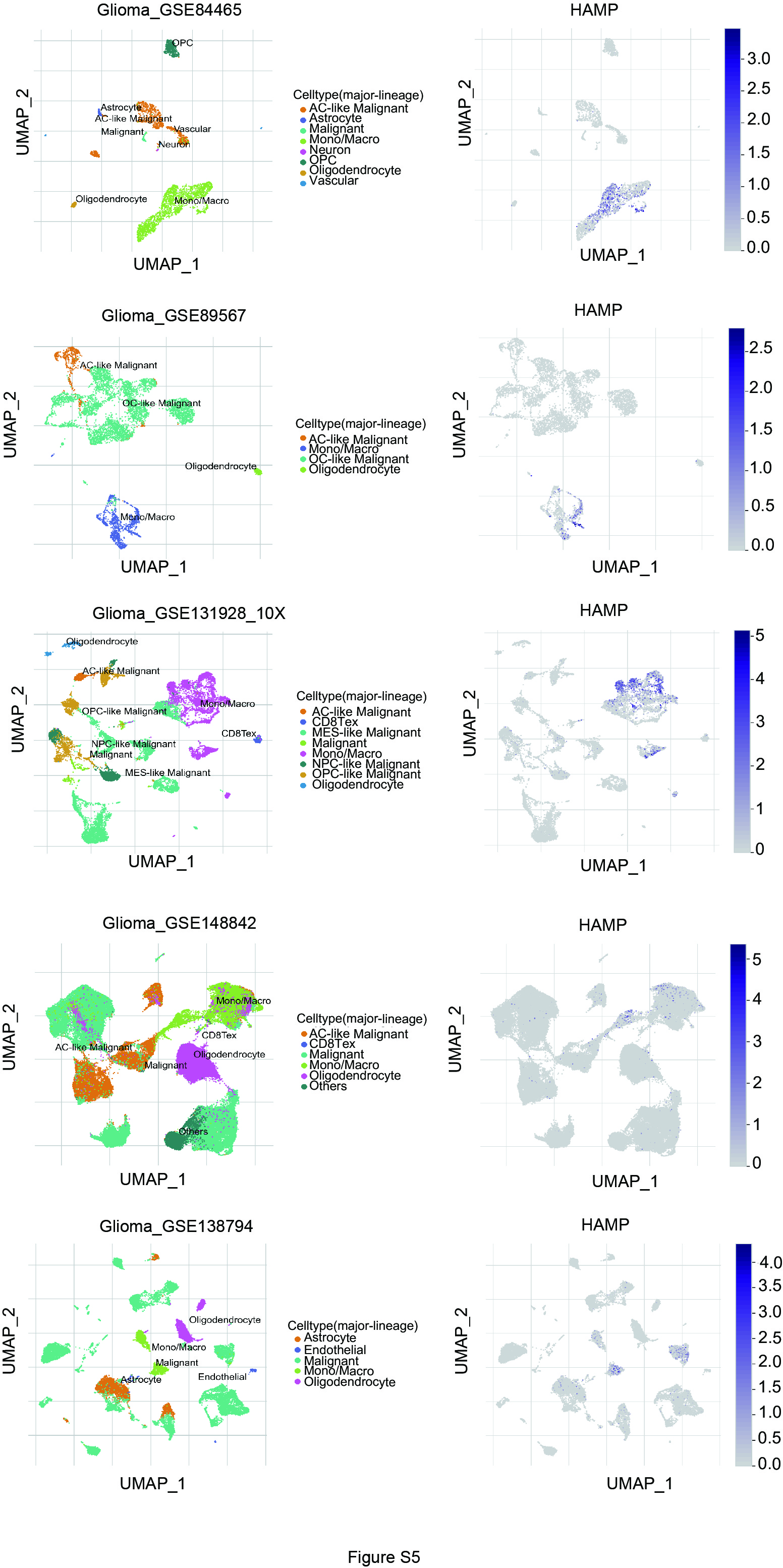

Supplement: Supplementary Figure 5 — Hepcidin expression in different cells was analyzed in GSE84465, GSE89567, GSE131928, GSE148842 and GSE138794 datasets using the TISCH database. [file Image_5.jpeg]

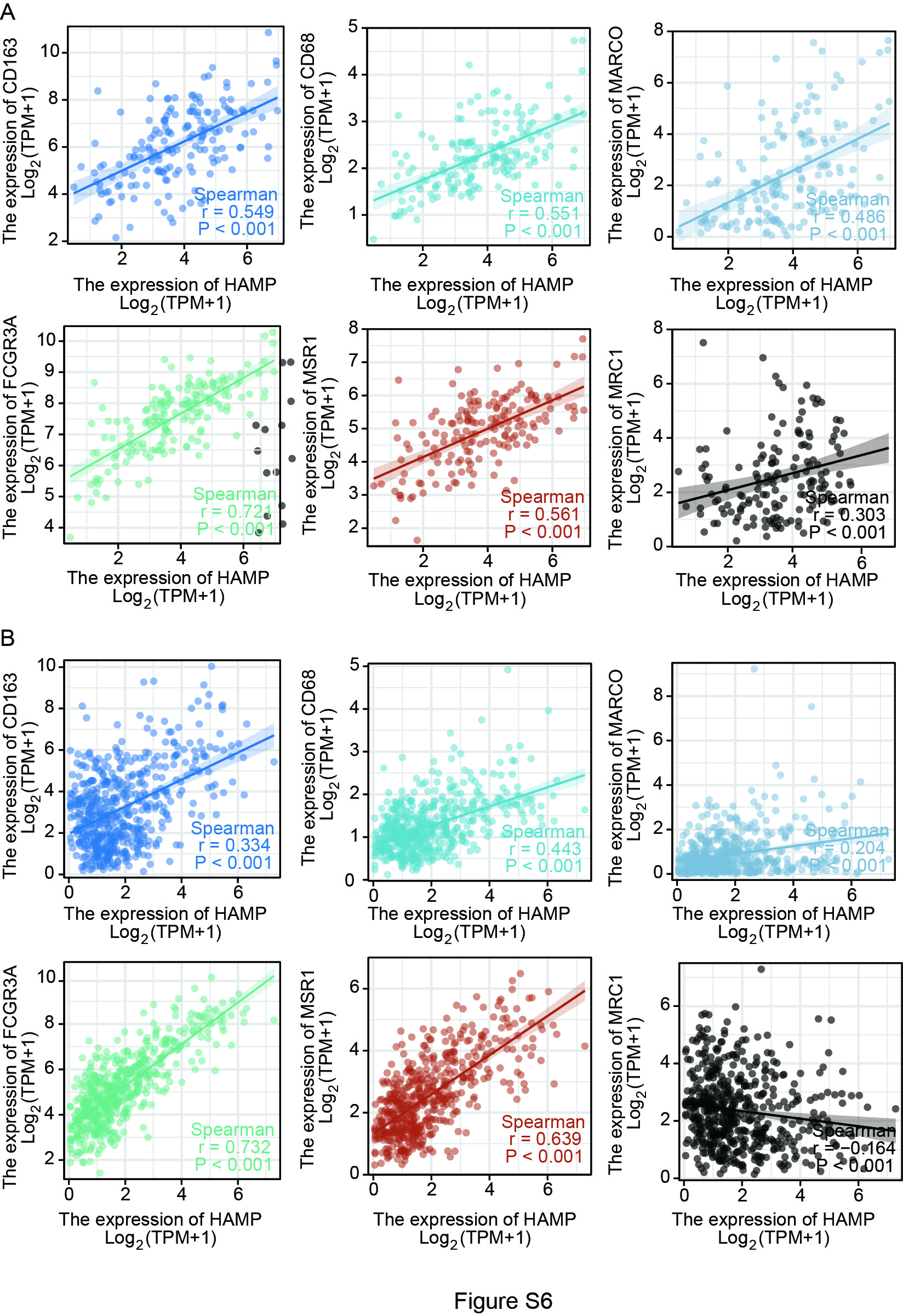

Supplement: Supplementary Figure 6 — The correlations between hepcidin and expression of macrophage markers based on the TCGA database. (A) Scatterplots of the correlations between hepcidin expression and CD163, CD68, MARCO, MRC1, MSR1 and FCGR3A expression in GBM. (B) Scatterplots of the correlations between hepcidin expression and CD163, CD68, MARCO, MRC1, MSR1 and FCGR3A expression in LGG. [file Image_6.jpeg]

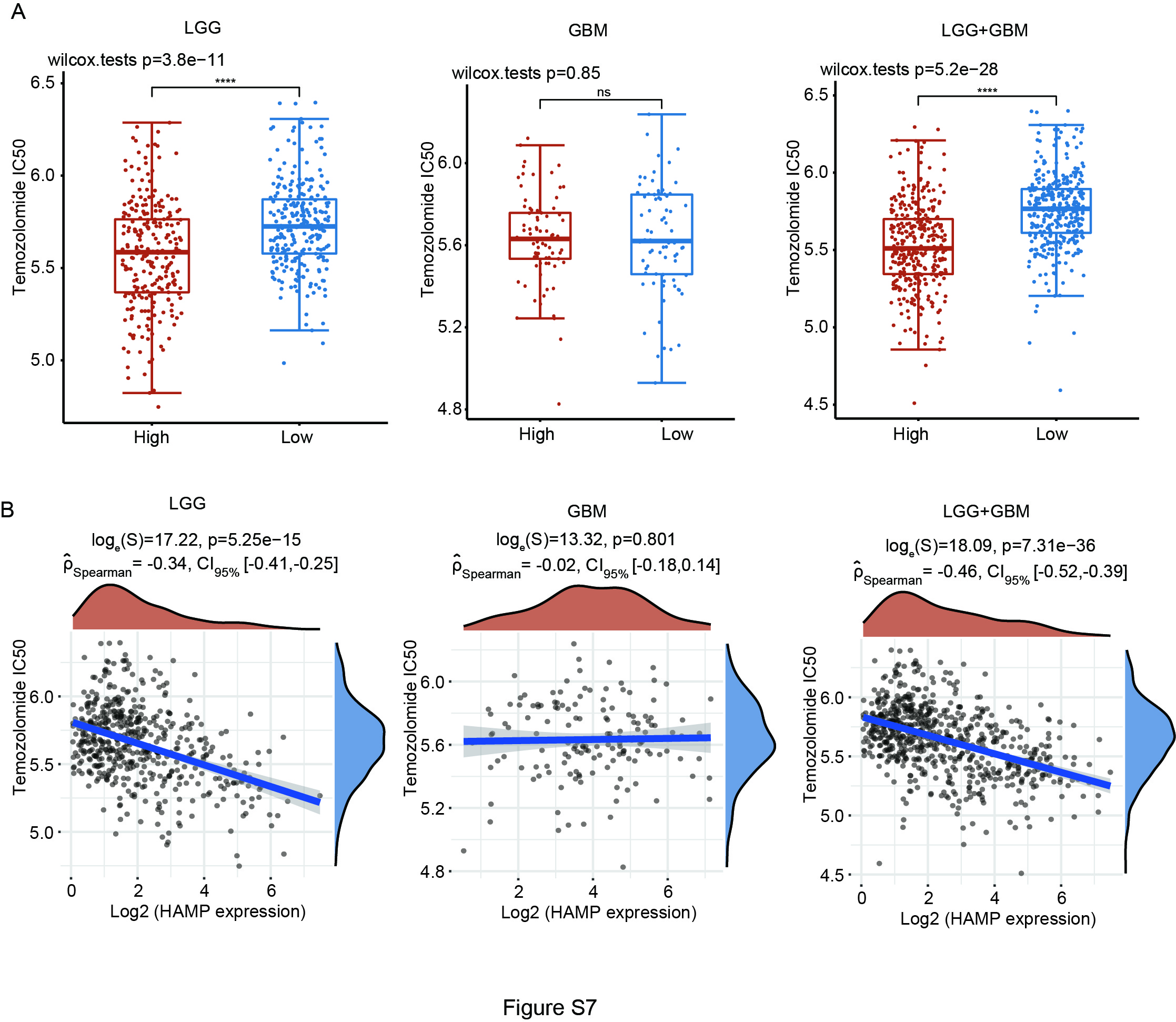

Supplement: Supplementary Figure 7 — The effect of hepcidin expression on temozolomide sensitivity. (A) The higher the expression of hepdicin, the lower the IC50 value of temozolomide in brain tumors. (B) Hepcidin expression was negatively correlated with temozolomide sensitivity in brain tumors. ****p < 0.0001. [file Image_7.jpeg]
